# Stigmatization attitudes of medical staff toward people with respiratory syndromes during COVID-19 pandemic

**DOI:** 10.1192/j.eurpsy.2021.787

**Published:** 2021-08-13

**Authors:** M. Sorokin, E. Kasyanov, G. Rukavishnikov, O. Makarevich, N. Neznanov, G. Mazo, N. Lutova

**Affiliations:** 1 The Integrative Pharmaco-psychotherapy Of Mental Disorders, V.M.Bekhterev National medical research center for psychiatry and neurology, Saint-Petersburg, Russian Federation; 2 Department Of Translation Psychiatry, V.M.Bekhterev National medical research center for psychiatry and neurology, Saint-Petersburg, Russian Federation

**Keywords:** distress, COVID-19, stress, Stigma

## Abstract

**Introduction:**

The health care workers have extremely high risks of adverse psychological reactions from COVID-19 pandemic. On the other hand, patients with respiratory syndromes face stigmatization due to their possible contagiousness of SARS-Cov-2.

**Objectives:**

To study the association of behavior, psychological distress in health care workers, and their stigmatization attitudes to the patients.

**Methods:**

The online-survey of 1800 health care workers performed during different lockdown periods in Russia: the first week and the last (30/Mar-5/Apr/20 and 4-10/May/20). The Psychological stress scale (PSM-25), modified Perceived devaluation-discrimination scale (Cronbach’s α=0.74) were used. Dispersion analysis with p-value=0.05 and Cohen’s d, Cramer’s V calculation (ES) performed.

**Results:**

In the 2nd phase medical stuff more often wore masks (64% vs. 89%; χ2=98.7, p=0.000, df=1; ES=0.23) and gloves (30% vs. 57%; χ2=57.6, p=0.000, df=1; ES=0.18), continued perform hand hygiene (94-95%) and physical distancing (73-74%), but was restricted in most effective protective measure: self-isolation (49% vs. 36%; χ2=16.0, p=0.000, df=1; ES=1.0). The psychological stress levels decreased in the 2nd phase (ES=0.13), while the stigma levels (ES=0.33) increased. Physicians experienced more stress compared with nurses and paramedical personnel (ES=0.34; 0.64) but were less likely to stigmatize SARS-CoV-2 infected individuals (ES=0.43; 0.41). The highest rates of contacts with COVID-19 patients (83%) were reported by physicians (χ2=123.0; p = 0.00, df=4; ES=0.28).

**Conclusions:**

Direct contact with SARS-Cov-2 is associated with a significant increase in stress among medical personnel. However, the stigmatizing reactions are not directly associated with the risks of infection and are most prevalent among nurses and paramedical personnel.

